# Notification of blood donors who test positive for transfusion‐transmissible infections

**DOI:** 10.1111/vox.13796

**Published:** 2025-01-14

**Authors:** Sheila F. O'Brien, Kiyuri Naicker, Lori Osmond, Kelly Holloway, Steven J. Drews, Mark Bigham, Mindy Goldman

**Affiliations:** ^1^ Epidemiology & Surveillance Canadian Blood Services Ottawa Ontario Canada; ^2^ School of Epidemiology & Public Health University of Ottawa Ottawa Ontario Canada; ^3^ Donation and Policy Studies Canadian Blood Services Ottawa Ontario Canada; ^4^ Microbiology Canadian Blood Services Ottawa Ontario Canada; ^5^ Department of Laboratory Medicine & Pathology, Faculty of Medicine & Dentistry University of Alberta Edmonton Alberta Canada; ^6^ Medical Laboratory and Stem Cell Services Canadian Blood Services Ottawa Ontario Canada; ^7^ Department of Pathology & Laboratory Medicine University of Ottawa Ottawa Ontario Canada

**Keywords:** blood donors, infectious disease testing, notification

## Abstract

**Background and Objectives:**

Despite screening procedures, a few blood donors confirm positive for transfusion‐transmissible infections and are deferred. Effective notification of laboratory results is essential to ensure that donors are advised of confirmed results and to seek medical care. Here we report results from post‐notification interviews of Canadian Blood Services donors.

**Materials and Methods:**

Over 17 years, 2006–2022, all donors with confirmed positive results for hepatitis B virus (HBV), hepatitis C virus (HCV), human T‐cell lymphotropic virus (HTLV) and syphilis were notified by registered mail of their result and advised to see a physician. In a separate communication, all donors were later invited to participate in a scripted interview asking whether they tested positive for an infection; if yes, which one, what their reaction was, whether they consulted a physician and whether public health contacted them. Frequencies of responses were calculated.

**Results:**

Of 2654 donors with confirmed positive test results, 876 (33%) participated; 90% said they were informed of a positive test result. Of these, about a quarter did not know for which infection they were positive. Most were surprised, and some were sad or disappointed. Most saw a physician after notification (77%). About two‐thirds with HBV or HCV said they were contacted by public health, slightly fewer (58%) with syphilis, 27% of those with HTLV.

**Conclusion:**

Most donors recalled being notified and were aware of their positive test, but details of the infection were sometimes not understood or recalled, and not all donors consulted a physician about the infection.

**Highlights**

- Donors are notified of confirmed positive tests by registered letter, and provincial/territorial public health departments are notified as required by law. The majority of donors who participated in the study were successfully notified that they had a confirmed positive transmissible disease test result.
- Most donors were surprised to learn that they had a confirmed positive transmissible disease test, suggesting that most donors were not aware of their infection before the notification of donor testing results.
- Not all donors with confirmed positive donation testing results chose to see a physician about their results.

## INTRODUCTION

Testing blood donations for markers of transfusion‐transmissible infections is foundational to blood safety. Even though donors are screened for infection risk factors prior to donation through a self‐administered donor health questionnaire, a small number of donors test positive for infectious markers every year [1]. Blood operators have both ethical and legal responsibilities to inform donors of their infection and to defer them [2]. In Canada, these responsibilities are outlined in Regulations and Standards, although the method of notification is at the discretion of the blood operator [3, 4].

It is also important that donors receive details about the infection and how to prevent transmission to others and are advised to consult a physician for follow‐up. In Canada, confirmed positive transmissible disease results for most blood‐borne infections are reported to provincial/territorial public health authorities as required by law.

Confirmatory testing of infection reactive donations is carried out primarily to counsel donors about the medical significance of their result, with secondary benefits being surveillance of blood safety, to be more informative for public health authorities and to incorporate into donor re‐entry protocols [1, 5, 6]. Similar to most high‐income countries, in Canada donors are notified of their positive test results by a standard letter tailored to a specific test result [7].

Despite the importance of donor notification, very little evaluation of this process has been reported [8]. The donor notification processes in 15 countries were described in a *Vox Sanguinis* International Forum in 2017 [7]. There are a few reports of surveys of donors notified of abnormal results such as in 1997 in the United States and in 2008 in the United Kingdom and a qualitative study [9, 10, 11]. There have been several studies reported from low‐ and middle‐income countries, but as most do not carry out confirmatory testing, these are not directly comparable to those from countries such as Canada [12].

As part of telephone interviews for risk factors, donors who had tested positive for infectious disease markers were asked about their experience with the notification process [13, 14]. Here, we report on data collected over the past 17 years (2006–2022) to describe the outcomes of the donor notification process at Canadian Blood Services (CBS).

## MATERIALS AND METHODS

### Donor notification process

All donors must provide government‐issued identification at the time they register to donate and provide a contact address. Prior to donating blood, all donors are informed that their blood will be tested for markers of infectious disease, that any abnormal results will be communicated to them and that public health authorities will be informed of confirmed positive test results, as required by law. At CBS, all donors with positive confirmatory results for any infectious disease marker are notified by registered letter of their result. In addition, donors positive for human immunodeficiency virus (HIV) and West Nile virus are notified by telephone, and at the discretion of a CBS medical officer, donors with other infections may also be contacted by telephone [15].

The notification letter and corresponding explanatory and supplementary information are specific to the test results. Generally, donors are informed that they are ‘very likely’ infected with the specific agent. The exceptions are when donors have lone hepatitis B virus surface antigen (HBsAg) positive or lone hepatitis B virus (HBV) Nucleic Acid Test (NAT) reactive results, in which case they are informed that they ‘may be’ infected (potentially false reactive results); with lone anti‐hepatitis C virus (HCV)‐positive results, they are told it is indicative of a past infection; if syphilis antibody‐positive, they are informed that they are ‘very likely’ infected or they may have had an infection in the past. The letter advises the donor to consult a physician and contains information about symptoms, health risks and preventing transmission. If he or she is a repeat donor, they will be told that previous donations will be investigated to see if a recipient may have been infected (lookback investigation) and that public health authorities will be informed as required by law. They are also provided with internet links to additional reliable information. A corresponding test‐specific template physician letter is also enclosed with the donor notification letter for the donor to take with them to a physician. Donors with lone antibodies to hepatitis B core antigen reactive results are informed by letter and indefinitely deferred but are not interviewed. The screening and confirmatory assays have been described previously [14, 16, 17].

### Study design

Since 2005, all donors with confirmed positive results for HBV, HCV, human T‐cell lymphotropic virus (HTLV; including HTLV‐I and HTLV‐II) or syphilis were invited to participate in a scripted interview after the notification process had been completed. HIV‐1/2 infections are rare in Canadian blood donors, and as their risk factors are collected at the time of telephone notification of their positive test, they are excluded. Donors with more than one infection are also rare and are not included. Confirmatory or supplementary testing is performed on most serological repeat‐reactive screening tests (not hepatitis B core antibody, for which there is no Health Canada‐licensed confirmatory assay). From 2005 to 2013, 12 weeks were allowed following a confirmed positive test donation before contacting donors, and thereafter 8 weeks. Donors were invited by letter to participate in an interview about the risk factors. A trained interviewer from a research company (Decision Point Research) then contacted the donor by telephone to arrange a time for an interview if the donor agreed. The donors were asked to ensure that they were in a private place at the time of the interview. The interview followed a scripted questionnaire (computer‐assisted telephone interview, CATI). The first set of questions asked the donor whether they had been told after their last donation that they had a positive test for an infectious disease. If donors answered affirmatively, they were asked which one, what their initial reaction to this news was, whether they consulted a physician and whether public health contacted them after they were informed. All questions were open ended. The donor's age, sex, donation status and region of residence were obtained from the CBS donor records to compare participants from non‐participants. The study period included all responses between 2006 and 2022. Donors with syphilis‐positive results were included from 2006 to March 2015, at which point interviews for donors with syphilis‐positive results were suspended; but it re‐started from 2020 to 2022 as part of assessment of new sexual behaviour‐based screening criteria [18]. The study was approved by the CBS Research Ethics Board.

### Analysis

Answers to questions were pre‐coded in the CATI except the question about the donor's reaction to being told that they had a positive test. This was generally quite brief and was coded to be able to group answers under common response categories, and a content analysis was performed to provide frequencies for each category.

Frequencies of demographic variables were calculated. Response frequencies were also calculated for each infection (HBV, HCV, HTLV and syphilis). Chi‐square tests were used to compare response rates by demographic groups using SAS version 9.4 (Cary, NC). To understand whether donors with test profiles that may not be indicative of current infection answered questions differently, as a secondary analysis, answers were compared in the following groups: those with lone HBsAg or lone HBV NAT versus other HBV positive; and, those with lone anti‐HCV versus anti‐HCV and HCV NAT.

## RESULTS

Over the 17 years of the study, there were 2654 donors notified of a positive HBV, HCV, HTLV or syphilis test who were invited to participate. Of these, 876 (33%) donors participated in the interview. Response rates by infection are shown in Table 1. The participation ranged from just over a third of those with HBV‐ or HCV‐positive results to about a quarter of those with HTLV‐ or syphilis‐positive results. The demographics of all donors with positive tests and those who participated in the interview are shown in Table 1. Donors with confirmed positive results were distributed evenly by age, except fewer in older donors, more likely to be male or first‐time donors and were distributed geographically proportional to collections. The proportion of donors who participated in the survey varied by demographic variables. Older donors were somewhat less likely to participate, as were first‐time donors and donors with syphilis or HTLV.

**TABLE 1:** Comparison of interview participants and non‐participants by demographic characteristics (2006–2022).

	Participants (*N* = 895)	Total invited (*N* = 2684)	Percentage of those invited who participated	*p*‐value
Age (years)				
<30	244 (27.3%)	596 (22.2%)	40.9	<0.0001
30–39	148 (16.5%)	509 (19.0%)	29.1	
40–49	183 (20.4%)	594 (22.1%)	30.8	
50–59	233 (26.0%)	682 (25.4%)	34.2	
≥60	87 (9.7%)	303 (11.3%)	28.7	
Sex				
Male	570 (63.7%)	1751 (65.2%)	32.6	0.2326
Female	325 (36.3%)	933 (34.8%)	34.8	
Positive marker				
HBV	369 (41.2%)	1078 (40.2%)	34.2	0.0023
HCV	346 (38.7%)	951 (35.4%)	36.4	
HTLV	43 (4.8%)	156 (5.8%)	27.6	
Syphilis	137 (15.3%)	499 (18.6%)	27.5	
Status				
First time	739 (82.6%)	2275 (84.8%)	32.5	0.0254
Repeat	156 (17.4%)	409 (15.2%)	38.1	
Region				
British Columbia	185 (20.7%)	597 (22.2%)	31.0	0.0258
Alberta	183 (20.4%)	522 (19.4%)	35.1	
Prairies	65 (7.3%)	227 (8.5%)	28.6	
Ontario	421 (47.0%)	1218 (45.4%)	34.6	
Atlantic	41 (4.6%)	120 (4.5%)	34.2	

Abbreviations: HBV, hepatitis B virus; HCV, hepatitis C virus; HTLV, human T‐cell lymphotropic virus.

**FIGURE 1:**
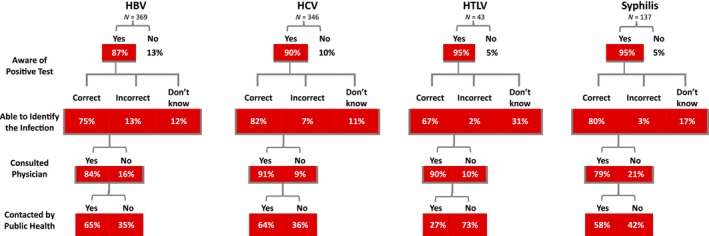
Breakdown of donor responses to questions by infectious disease marker. All donors interviewed were sent notification of confirmed infectious diseases tests by registered mail, although some said they were not aware. Of those who were aware, about a quarter did not know which infection they were positive for, many consulted a physician and fewer said they were contacted by public health. Assuming those who said they were not aware of testing positive did not consult a physician, combined with those who were aware but did not, about 75% of donors who were notified consulted a physician. Each level in the figure is comprised of all donors who said they were aware they had a positive test for an infectious disease. HBV, hepatitis B virus; HCV, hepatitis C virus; HTLV, human T‐cell lymphotropic virus.

Of the 876 donors who participated in the interview, 786 (90%) said that they had been informed of their positive test result (see Figure 1). However, about a quarter stated that either their infection was something other than what it was or they did not know which infection they had been notified of. Most donors went to a physician after being notified (80%–90% by infection). About two‐thirds of donors with HBV or HCV said they were contacted by public health, slightly fewer (58%) with syphilis and only 27% of those with HTLV. The donors with HTLV who said they were contacted by public health were from British Columbia (BC) (6) and Ontario (3). Data were missing from questions as follows: able to identify infection (13.1%); consulted physician (0.1%); contacted by public health (15.5%).

Of the 369 donors with HBV‐positive results, there were 5 with lone HBV NAT‐ and 59 with lone HBsAg‐positive results (*N* = 64 in total) compared with 305 with at least two of three HBV markers positive. Those with lone HBV results appeared slightly less likely to be aware that they had a positive result compared with those with more than one positive marker, although not statistically significant (78% vs. 86%, *p* = 0.07). Of those who were aware, those with lone positive results were less likely to correctly state that it was HBV (59% vs. 78%, *p* = 0.02) but not less likely to consult their physician (76% vs. 86%, *p* = 0.09) and equally likely to remember being informed by public health (54% vs. 64%, *p* = 0.3). There were 102 donors interviewed with lone anti‐HCV‐positive results compared with 244 with anti‐HCV‐ and HCV NAT‐positive results. Those with lone anti‐HCV results were less likely to say that they had a positive test (76% vs. 93%, *p* < 0.0001). Of those who did, those with lone results less frequently stated that they were positive for HCV (74% vs. 85%, *p* = 0.049), somewhat fewer consulted a physician (83% vs. 93%, *p* = 0.01) but were equally likely to remember being contacted by public health (58% vs. 63%, *p* = 0.5). When all 876 donors with positive test results who participated, including those who said they did not have a positive infection result are considered, 678 (77%) said they had consulted a physician about their positive test.

When donors were asked what their reaction had been to learning that they had a positive blood transmissible infection result, most were surprised and some were sad and disappointed. The responses to this question are summarized in Table 2.

**TABLE 2:** Donor responses to ‘What was your reaction?’ to notification letter (*N* = 660).

Category^a^	Number (%)	Examples
Shock/surprise	348 (53%)	‘I was shocked and a bit surprised’. ‘Surprised because I had donated in the past’ ‘I took a hepatitis vaccine before donating and did not remember at the time of donating and I was surprised when I was informed of it’
Upset/sadness/anger/disappointment/not good	72 (11%)	‘Angry’ ‘I was a little bit sad’ ‘I was devastated and sad about the result’ ‘I feel like my donation didn't really matter’
Concern/fear	42 (6%)	‘Scared’ ‘I was concerned’ ‘Anxious’
Disbelief/confusion	42 (6%)	‘Thought it was a false positive’ ‘I thought it was a false positive—I'm doing regular exams, I was not concerned about the results’ ‘Confused. Didn't know what exactly was going on. More information would have been appreciated’ ‘I understand the letter but I can't understand the test results’
Not concerned	29 (4%)	‘I didn't feel anything—No reaction. I read the letter and it didn't make me upset. My thinking is that if I had it for so long and I didn't know about it, then I don't know, and I feel fine’ ‘No reaction because I went through a hepatitis C programme’
Some awareness	21 (3%)	‘I had it 3 years ago and I had gotten 2 shots for it so I thought it had gone away’. ‘I already knew that I had it before. However, I was a little bit upset that I couldn't donate anymore because I didn't have the hepatitis’. ‘My blood is a universal blood type, so would like to donate again’. ‘I knew I didn't have it at time of donation; but I know I have had it some time ago. I had no reaction because I had it at one point over 12 months ago’
Wondered how they contracted/sought information	18 (3%)	‘Started looking up more information and if there was a cure’ ‘Spoke to my doctor’ ‘Did not understand how I contracted it’
Other	24 (4%)	

^a^ More than one category was possible.

## DISCUSSION

Over 17 years of surveying donors notified of positive test results for HBV, HCV, HTLV and syphilis, we report that after being notified by the blood service, most donors in the study were aware that they had tested positive for a blood transmissible infection, although not always able to correctly identify which one. About three‐quarters followed the advice in the letter to consult a physician. Fewer said that they had been contacted by public health.

Notifying donors of a positive result for a blood‐borne infection is important to direct the donor to a physician and to make the donor aware of the potential risk of infecting others. Our results indicate that after notification most donors were aware that they had a positive test, but not all. As the letter was sent by registered mail to the donor's home, and as we were able to contact the donor afterwards, the notification letter should have been received, although this could not be verified. That some donors denied knowing they had a positive test is perplexing. The letter may have been received by someone else in the household and never reached the donor. The donor may not have understood it or was in denial. It is possible that the donor was aware but did not want to admit to the interviewer because there is stigma associated with blood‐borne infections.

That some donors said they were notified but were unsure of which infection suggests imperfect communication. Limited understanding of test results is also a common problem in communicating patient test results in the clinical environment [19]. It appears that some donors may simply be unable to remember the name of the infection but be aware of what it is. For example, a few donors who could not name their HBV or HCV infection as such said they had hepatitis. In addition, donors may not have felt comfortable admitting which infection they had. There were nuances in communication to the donor by test result, which likely affected the way they responded. For example, donors with lone anti‐HCV results were less likely to say they had a positive test. Most often, a lone anti‐HCV result indicates past infection that has resolved, either spontaneously or following antiviral treatment [20, 21]. Donors with this result may have understood they were not infected and interpreted the question as asking if they have an active infection rather than simply a positive test because they were asked about hepatitis in the last 6 months in the donor history questionnaire.

Of donors who said they had a positive test, nearly 90% said they consulted a physician. However, if we assume that those who denied having a positive test also did not see a physician, only about three‐quarters of all donors interviewed consulted a physician. There are valid reasons why a donor may not consult a physician. Some may have already known and been treated, such as for syphilis [22]. In pre‐donation screening, donors were asked if they had syphilis in the last year, but if it was more than a year ago they were eligible to donate. Those who had a positive test for anti‐HCV but not HCV NAT may have known they no longer had an active infection. It is possible that some donors may not equate certain scenarios with seeing physician. For example, they may have consulted a health professional by telephone, visited an emergency department or attended a specialty clinic (e.g., for sexually transmitted infections).

Local public health officials (usually, a public health nurse) will attempt to contact the donor to ensure they are informed of their positive test, to advise them of risks to themselves and to others and to initiate the cascade of health care. The percentage of donors who said they were notified by public health is low: about two‐thirds. All infections were notifiable in all provinces, except HTLV which is notifiable only in Saskatchewan and BC (plus the territories where blood is not collected). HTLV is generally not included in infectious disease screening programmes other than for blood donation. Most HTLV‐positive donors who said public health notified them were living in BC but there were three living in Ontario who may have misunderstood who was meant by ‘public health’. This may suggest that others could also have been unclear of the meaning of the term ‘public health’ that was used in the follow‐up donor questionnaire. However, that more donors with HTLV said they were not contacted by public health than other markers in those jurisdictions where all donors should have been contacted suggests that donors often answered the question appropriately. The term ‘public health’ could be confusing because the person who contacts the donor may not have identified themselves as being from ‘public health’. Public health follow‐up is managed differently in different provinces and can vary by region within a province. The follow‐up could be managed by specialty units such as hepatology clinics or sexually transmitted infection clinics who may identify themselves as being from their specialty clinic. The extent of follow‐up is also variable by region such that it could be simply informing the donor of their test result and risk to others, whereas in some regions there may be help arranging an appointment with a caregiver. There may be situations where public health would decide not to contact a donor, such as when they have evaluated the test results and concluded that the donor was not infected. Table 3 lists some reasons why donors may have said they were not contacted by public health.

**TABLE 3:** Possible reasons for saying not contacted by public health.

Reason	Explanation
Their healthcare provider may have been in touch with public health	If the donor is under care, the regional public health unit may not be required to follow up.
The agent may not have been from ‘public health’ per se	Some regions will hand over contact to a relevant care provider such as the ‘sexually transmitted diseases unit’ or a hepatology unit. The donor may not understand the public health activity behind this.
The test results may indicate a resolved infection	For hepatitis C and antibody‐positive NAT‐negative donation or for syphilis a low rapid plasma reagin/non‐reactive result would suggest resolved past infection. Contacting these individuals may not be prioritized by all public health regions.
The infection may not be notifiable	HBV, HCV and syphilis are reportable in all jurisdictions; however, HTLV is notifiable only in Saskatchewan and two northern territories.

Abbreviations: HBV, hepatitis B virus; HCV, hepatitis C virus; HTLV, human T‐cell lymphotropic virus.

Our study has some important limitations. About a third of donors with infectious disease positive results participated in the interview and are not necessarily representative of those who did not. The interviews were carried out by trained interviewers from a research company using a scripted questionnaire. This ensured that each question was asked in the same way to each donor but did not allow the in‐depth probing of a qualitative interview; also, more detailed information that may have improved interpretation of donor responses was not collected.

Overall, our results indicate that after being notified by the blood service, most donors are aware that they had tested positive for an infectious marker, and many, but not all, consulted a physician. Qualitative interviews with donors post notification may be helpful to identify specific actions that could be taken to improve notification and subsequent action. Although all donors are notified by registered letter (sometimes also by telephone) as per standard operating procedures, and public health is notified of all confirmed positive tests as required by law, the donor‐centric clinical outcome is unknown. It appears that some donors—for a variety of reasons—do not follow the recommended advice to consult a physician. A potential area of further enquiry arising from this analysis is to collaboratively explore with provincial public health authorities whether there are opportunities to increase the proportion of donor follow‐up after public health notification of a confirmed positive test result for a reportable disease.

## CONFLICT OF INTEREST STATEMENT

The authors declare no conflicts of interest.

## Data Availability

The data that support the findings of this study are available on request from the corresponding author. The data are not publicly available due to privacy or ethical restrictions.
